# Whole-genome sequence of a marine bacterial strain, FRT2, belonging to the genus *Leeuwenhoekiella*, isolated from the seawater of the Obama Bay in Fukui, Japan

**DOI:** 10.1128/mra.00277-24

**Published:** 2024-05-02

**Authors:** Yuka Machii, Mao Tsukamoto, Takafumi Kataoka, Ryuji Kondo

**Affiliations:** 1 Department of Marine Science and Technology, Fukui Prefectural University, Obama, Fukui, Japan; California State University San Marcos, San Marcos, California, USA

**Keywords:** *Leeuwenhoekiella*, novel species

## Abstract

A marine bacterial strain, FRT2, was isolated from the surface water of the Obama Bay in Fukui, Japan. Its complete genome comprises one circular chromosome (3,806,097 bp), harboring 3,269 predicted protein- and 44-tRNA-encoding genes and 3 rRNA operons.

## ANNOUNCEMENT

The Department of Marine Science and Technology, Fukui Prefectural University, offers a variety of practical training as an introductory education for undergraduate students. Our microbiology group has attempted to isolate novel species of prokaryotes from aquatic environments. In the course of isolation, a yellow-orange-pigmented bacterium on 1/10-diluted Marine Agar 2216 (BD Difco), strain FRT2 was isolated from surface water of the Nishizu fishing port in Obama Bay in Fukui, Japan (35°30′50.2″N 135°44′55.3″E), on 22 July 2023. The results of preliminary PCR-amplicon sequence analysis targeting the V3/V4 region of the 16S rRNA gene indicated that closest relative of strain FRT2 was *Leeuwenhoekiella nanhaiensis* G18^T^ (NR_144589) and *Leeuwenhoekiella palythoae* DSM 19859^T^ (NR_104514) with 16S rRNA gene sequence identities of 94.49% and 95.12%, respectively, suggesting that the strain represents a novel species of the genus *Leeuwenhoekiella*, according to species delineation (1).

Strain FRT2 stored at −80°C with 25% (vol/vol) glycerol was inoculated into Marine Broth 2216 (BD Difco) and incubated at 20°C in the dark. Then, the cell pellet was harvested by centrifugation at 3,310 × *g* and 4°C for 30 min. Genomic DNA (gDNA) was extracted from the cell pellet by the standard phenol-chloroform method, as described previously (2). After the final purification of the extracted DNA using a short-read eliminator kit (Circulomics), the gDNA was sheared to 10- to 20-kb fragments using g-TUBE devise (Covaris). A long-read DNA library was constructed for strain FRT2 using the SMRTbell Express template prep kit v.2.0 (PacBio), following the manufacturer’s instructions, and sequenced using the PacBio Sequel IIe system. High-fidelity (Hi-Fi) reads were obtained from the subreads generated using circular consensus sequencing via SMRT Link v.10.1.00.119528. Of the 19,098 Hi-Fi reads containing >3,000 bp were screened using Filtlong v.0.2.1 (https://github.com/rrwick/Filtlong), and the remaining 15,290 reads were used for *de novo* assembly performed with the Flye v.2.9.2 (3) with polishing time of two, read error of 0.01, and assembly type of meta. Default parameters were used for all software unless otherwise specified. This resulted in a single 3,806,097-bp circular genome with a 39× coverage (*N*_50_ = 3,806,097 bp) and an overall G + C content of 39.0% as determined using DFAST (4), available at the DNA Data Bank of Japan. The genome was annotated using DFAST (4). The complete genome contained three ribosomal RNA operons, 44 tRNA genes, and a total of 3,269 coding DNA sequences.

In order to check whether strain FRT2 is a novel species according to bacterial criteria (5), digital DNA-DNA hybridization (dDDH), and average nucleotide identity (ANI) values were calculated using formula 2 of the Genome-to-Genome Distance Calculator tool (http://ggdc.dsmz.de/distcalc2.php) (6) and CJ Bioscience’s online ANI calculator (https://www.ezbiocloud.net/tools/ani) (7), respectively. The dDDH and ANI values of strain FRT2 to the type strains of *Leeuwenhoekiella* sp. with publicly available genome information were lower than the thresholds (70% for dDDH and 95% for ANI) for species delineation (5), indicating that strain FRT2 is a novel species of the genus *Leeuwenhoekiella*.

## Data Availability

The genome sequence of strain FRT2 has been deposited in DNA Data Bank of Japan (DDBJ) under the accession number AP029251. The raw reads have been deposited in the DDBJ Sequence Read Archive under the accession number DRR530218. The BioProject and BioSample accession numbers are PRJDB17504 and SAMD00738033, respectively.
